# Caring for infants is associated with increased reproductive success for male mountain gorillas

**DOI:** 10.1038/s41598-018-33380-4

**Published:** 2018-10-15

**Authors:** Stacy Rosenbaum, Linda Vigilant, Christopher W. Kuzawa, Tara S. Stoinski

**Affiliations:** 1 Department of Anthropology, Northwestern University, Evanston, IL USA; 2 Davee Center for Epidemiology and Endocrinology, Lincoln Park Zoo, Chicago, IL USA; 3 Max Planck Institute for Evolutionary Anthropology, Leipzig, Germany; 4 Institute for Policy Research, Northwestern University, Evanston, IL USA; 5 The Dian Fossey Gorilla Fund, Atlanta, GA USA

## Abstract

Socioecological theory predicts that male parenting among mammals should be rare due to the large payoffs of prioritizing mating effort over parenting. Although these predictions are generally met, in some promiscuous primate species males overcome this by identifying their offspring, and providing benefits such as protection and resource access. Mountain gorillas, which often organize into multi-male groups, are an intriguing exception. Males frequently affiliate with infants despite not discriminating their own from other males’ offspring, raising questions about the function of this behavior. Here we demonstrate that, independent of multiple controls for rank, age, and siring opportunities, male gorillas who affiliated more with all infants, not only their own, sired more offspring than males who affiliated less with young. Predictive margins indicate males in the top affiliation tertile can expect to sire approximately five times more infants than males in the bottom tertile, across the course of their reproductive careers. These findings establish a link between males’ fitness and their associations with infants in the absence of kin discrimination or high paternity certainty, and suggest a strategy by which selection could generate more involved male parenting among non-monogamous species.

## Introduction

Social relationships between adult males and infants are quite rare among group living mammals, due to the relatively high payoffs for males of investing in mating rather than parenting^1^. However, they are observed in some Old World primates, including baboons, macaques, gorillas, and humans^2–8^. Such relationships appear to have fitness-relevant benefits for infants, such as improved access to resources, and protection from infanticide, predation, and conspecific harassment^6,9–11^.

Males themselves may benefit from these relationships in two ways. First, they may selectively interact with their current offspring, thereby engaging in paternal care. Evidence for the paternal care hypothesis is now well-established in some of the Old World primate species in which relationships between males and infants are common^7,9–14^). Alternatively, males may be investing in their future reproductive success, rather than their current offspring, if affiliating with infants improves their chances of siring infants’ mothers’ future offspring (i.e. the mating effort hypothesis^2,15–17^).

While the mating effort hypothesis has been a source of much speculation, empirical evidence for it has been sparse. Studies have depended on indirect proxies such as mating behavior, males’ choice of infant social partner(s), or the timing of males’ care behaviors relative to mating opportunities^2,3,5,9,17–19^. While informative, these tests rely on assumptions that are potentially problematic. For example, mating behavior may not translate into reproductive success when females mate promiscuously during estrus windows, while care behaviors could serve as a much broader signal to all available females rather than solely to a specific female (e.g.^16^). Furthermore, it is difficult to discern the influence of mating effort when males selectively direct paternal care to their own offspring. Since the two explanations are not necessarily mutually exclusive^17,20^, paternal care may disguise any mating effort effects.

Mountain gorillas are an ideal species in which to test whether caring for infants may be a form of male mating effort. Male-infant relationships are a prominent and important feature of mountain gorilla (*Gorilla beringei*) social groups, even when infants live in groups with multiple potential fathers^3,4,21^. Although the modal mountain gorilla group composition is one male, one or more females, and their offspring, some 40% of mountain gorilla groups contain >1 adult male, and some have been observed with up to 9 simultaneously co-resident males of siring age^22,23^ along with females and offspring. Dominant males sire the majority of infants, but other males regularly reproduce, and there can be considerable temporal variation in the degree of reproductive skew^24–26^. The most important type of care male gorillas are believed to offer infants is protection. In this species, sexually selected infanticide committed by extra-group (and very rarely, in-group) males is responsible for ~20% of overall infant mortality, though during periods of social instability it has risen as high as 37% (Karisoke Research Center long-term records^27,28^). Alongside this critical form of indirect care, male and infant mountain gorillas regularly affiliate directly with one another. Unlike some other Old World primate species in which there is evidence for various forms of paternal care, in this species interactions do not appear to be dependent on kin discrimination: males affiliate with infants irrespective of their relatedness^24^. This raises the question of what other function(s) these relationships might serve, and provides an ideal opportunity to determine whether males’ relationships with infants are related to their future reproductive success.

We used behavioral and genetic paternity data collected on wild mountain gorillas monitored by the Dian Fossey Gorilla Fund’s Karisoke Research Center since 1967, to test the hypothesis that male mountain gorillas who spend more time affiliating with infants have higher long-term reproductive success. If so, this would be consistent with male-infant relationships serving as a form of male mating effort, and could thereby help explain male-infant affiliation in a species characterized by paternity uncertainty, no apparent paternal kin discrimination, and facultative (rather than obligatory) male care.

## Results

**Figure 1:**
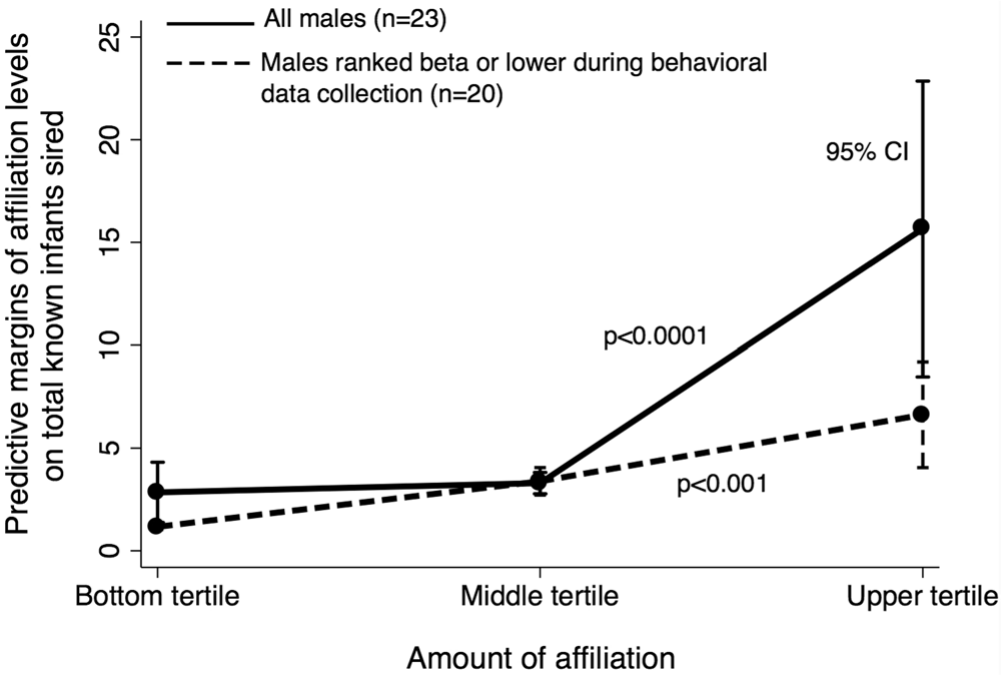
Males who affiliated more with infants in 2003 and 2004 sired more infants by 2014. Y axis is the total sired infants for males at a given affiliation level, calculated from predictive margins of the affiliation variable in a negative binomial regression model that controlled for males’ dominance rank and age at behavioral data collection and at the point they exited the dataset, as well as the number of siring opportunities they had (Table 1, also see methods). Affiliation values are a composite measure of the percent of total observation time males spent resting in contact and grooming with all available infants.

In a model that contained controls for the age and rank males achieved in their lives, the age and rank they held as of behavioral data collection, and adjusted for possible siring opportunities (see methods), we found that males who spent more time affiliating (i.e. grooming and resting in contact: see methods) with infants sired more offspring than their counterparts who spent less time affiliating with infants (Fig. 1, Table 1). Examination of predictive margins indicated that males in the middle tertile of affiliation could expect to sire 1.16 times as many infants over the course of their reproductive career as their counterparts in the bottom tertile, while males in the top tertile could expect to sire 5.50 times as many (Fig. 1). To determine whether this association was specific to affiliation, rather than a byproduct of male proximity to infants and their mothers, we ran the same model, but substituted the proportion of time males had at least one infant <2 m away for our affiliation variable. There was no relationship between proximity to infants and males’ reproductive success (ß = 1.74, SE = 2.25, p = 0.439, n = 23).

**Table 1:** Negative binomial regression model linking reproductive success and male-infant affiliation.

Predictor	Coef+/−SE	Z	P	95% CI
Affiliation	10.07+/−0.73	13.74	**0.000**	8.64, 11.51
Age at behavioral data collection	−0.10+/−0.02	−5.09	**0.000**	−0.14, −0.06
Age when exited dataset	−0.01+/−0.02	−0.55	0.579	−0.05, 0.03
Rank at behavioral data collection (reference = alpha)				
Beta	−0.85+/−0.12	−6.88	**0.000**	−1.09, −0.61
Gamma	−1.54+/−0.39	−3.9	**0.000**	−2.31, −0.76
Subordinate	−3.03+/−0.47	−6.41	**0.000**	−3.95, −2.10
Highest lifetime rank achieved (reference = alpha)				
Beta	1.36+/−0.12	11.65	**0.000**	1.13, 1.59
Gamma	−0.77+/−0.25	−3.12	**0.002**	−1.26, −0.29
Subordinate	−20.97+/−0.75	−27.92	**0.000**	−22.44, −19.50
Constant	1.42+/−0.92	1.54	0.123	−0.38, 3.22

n = 23 males, 109 infants; pseudo R squared = 0.482. Outcome variable is the total number of known infants males sired by 2014. Affiliation predictor is a composite measure of the time males spent grooming and resting in contact with all available infants during behavioral data collection in 2003–04. Relevant age and dominance rank variables are included as controls, along with reproductive opportunity as an exposure variable. Results confirm previously established relationships among male rank, age, and reproductive success in the study population.

Finally, we re-ran the original model, but included only the males who were beta- or lower-ranked during behavioral data collection. Again, there was a strong, statistically significant relationship between affiliation and reproductive success (β = 8.49, SE = 2.48, z = 3.42, p = 0.001, n = 20 males & 52 infants) (Fig. 1). Predictive margins were very similar to the model containing the full complement of males, though the effect was more linear. Males in the middle tertile of affiliation could expect to sire 2.90 times as many infants over the course of their reproductive careers as the males in the bottom tertile, while males in the top tertile could expect to sire 5.65 times as many infants (Fig. 1).

## Discussion

Previous research on promiscuous species has assumed fitness benefits to males who protect or provide resource access to their genetic offspring^6,9–12^, but to our knowledge our study is the first to directly link male-infant affiliation to male reproductive success in a promiscuous mammal. This relationship is consistent across male dominance ranks, suggesting that affiliating with infants is a generalized reproductive tactic, rather than an alternative strategy used only by males who are less successful at competing for high rank and its associated reproductive benefits.

While our data cannot directly address the mechanism by which the observed correlation between affiliation and reproductive success occurs, results are consistent with the mating effort hypothesis^2^. Females may preferentially mate with males who interact most with, or are especially tolerant of, infants. So far as we are aware, our data represent the most direct evidence for it to date, since other tests have relied on behavioral proxies rather than actual reproductive output^2,3,5,9,17–19^. Given the different methodologies, it is difficult to determine whether this means that mating effort applies in gorillas but not in other primate species, or whether other species would have similar results if analogous data were available.

One serious methodological limitation is the difficulty of detecting mating effort in species in which, unlike mountain gorillas, males selectively direct paternal care to their own offspring (e.g.^10,12^). Since the two explanations do not have to be mutually exclusive^17,20^, paternal care could disguise any mating effort effects. There is evidence that females prioritize males who provide care among non-monogamous birds, spiders, and fish^29–31^, and in humans, women rate men who have a higher affinity for children as better long-term mate prospects than men who are less interested in children^32^. Though it is not clear why females might prefer males who affiliate with infants in species in which male care is facultative rather than obligatory, one possibility is that males’ behavior is an honest signal of intention to provide protection and defend resource access when the need arises (e.g.^9^). Future work should explore this possibility, beginning with infant survival data. Karisoke’s extensive long-term demographic record can provide important information about infant outcomes that may shed light on female (or male) motivation.

Although our data are consistent with the mating effort hypothesis, alternative explanations are also possible. One potential scenario is reverse causality—that is, males who sire more infants interact more with infants. Although this possibility cannot be entirely ruled out, there are three reasons we believe our data are less consistent with this interpretation. First, in exploratory analyses, there was no indication that males who had more infants present at the time of behavioral data collection were more likely to affiliate with infants, which should have occurred if this was the case (i.e., males who had more offspring available did not do more ‘fathering’). Second, the result held even with two controls in place for male rank, and when the sample was limited to males who held ranks other than the dominant position during behavioral data collection. Since dominant males are more likely to sire infants than non-dominant males, we would expect the result to be weaker or to disappear when adjusting for male dominance, which it did not. Finally, the paternity data go forward in time relative to the behavioral data. Some males had just commenced their reproductive careers, suggesting the behavioral pattern may be in place before males have offspring of their own.

Another possibility is that males who are generally more gregarious or socially competent, and therefore interact the most with infants, are also more successful at obtaining mating opportunities and/or less likely to be marginalized or evicted from their social group (but see also^23^). In this scenario, females would be attracted to males on the basis of traits like personality type rather than potential benefits to future offspring. Although multi-species meta-analyses have not found a consistent link between personality traits and reproductive success^33^, the one published study on personality in mountain gorillas did find an association between dominance, which strongly predicts reproductive success in this species, and personality dimensions corresponding to openness, sociability, and agreeableness^34^. If males’ reproductive success, dominance, and personality traits are all correlated, this poses challenges to any attempts to tease apart the specific motivations that drive female mate preference. It also highlights the need for additional research on the role that female mate choice may play in driving social behavior in primates, since it suggests that female choice could play a role that is as evolutionarily consequential as (e.g.) mating system or social group structure.

Regardless of the underlying mechanism, the tendency of males who directly affiliate with infants to have higher reproductive success points to an evolutionary pathway by which selection could catalyze the evolution of more costly forms of male parenting behavior. The relationship between the two variables does not need to be causal to have evolutionary implications. Even if males’ affiliation with infants is simply an epiphenomenon of selection on personality or other traits, the corresponding increase in male-infant interaction could drive selection on the nature of the interaction itself.

The potential for novel selective pressures is particularly salient in a species like mountain gorillas, who may only recently have begun living in social groups that facilitate a connection between males’ behavior towards infants and their reproductive success. Although our research has shown that males routinely affiliate with offspring that are not their own^24^, this may be a relatively recent phenomenon. Despite regularly organizing into multi-male groups, several lines of evidence point to single-male, multi-female groups as the likely ancestral social organization among mountain gorillas. They have morphological hallmarks of a species in which there was strong selection on males to engage in direct contest competition, including their unusually pronounced sexual dimorphism^35^, well-developed weaponry, and lack of adaptations to sperm competition such as large testes-to-body size ratio or fast-swimming sperm^36^. Additionally, closely-related western lowland gorillas, who are believed to have split from mountain gorillas ~1.75 mya^37^, live almost exclusively in single-male, multi-female groups^28,38^. Finally, early historical accounts of mountain gorilla groups in the Virunga Massif primarily describe single-male, multi-female groups^39,40^. Groups with multiple (3+) males and high male:female ratios, where paternity certainty is presumably lower^24,26,41^, were not regularly observed until the 1990s and early 2000s^22,42^.

Based on these multiple lines of evidence, it seems plausible that mountain gorillas only recently began living in multi-male, multi-female groups, where males faced meaningful paternity uncertainty. In some primate species females regularly mate with extra-group males (reviewed in^43^), but behavioral data suggest, and genetic paternity data confirm, that extra-group paternity is extremely rare in both mountain and western lowland gorillas^26,44^. Mountain gorillas’ apparent lack of paternal kin discrimination^24^ may be a byproduct of an evolutionary history in which co-residence was sufficient for males to identify offspring, resulting in the current pattern of ‘misdirected’ paternal care once their social group structure shifted^24^.

If a substantial proportion of the population lives in what could be an evolutionarily novel mating system, these indiscriminate relationships between males and infants may have novel evolutionary consequences. If such relationships carry costs for males^45^, existing socioecological theory leads to the prediction that a discrimination mechanism should now evolve, or that rates of interaction between males and infants should fall. In contrast to these expectations, our data provide evidence that male-infant interactions could be actively selected for in the absence of discrimination. This is a concrete example of how behavioral flexibility, in this case shifts in social group structure, may generate new phenotypic variation on which selection can then act^46^.

Though the potential evolutionary implications of behavioral flexibility are extensive, in this case it has specific revelevance for our understanding of the evolution of paternal care. These animals’ behavior, and its relationship to their reproductive success, point to one potential path by which elaborate forms of male investment might evolve in the absence of high paternity certainty. Across taxa, male caretaking is more often associated with monogamy than with any other mating system^47,48^. Phylogenetic analyses suggest that mammalian monogamy often evolves as a form of mate guarding among species in which breeding females are solitary and intolerant of one another, with paternal care following suit^48^. However, this does not explain the unusual cases where male caretaking occurs outside the context of monogamy, and in particular among species wherein females clearly benefit from aggregating.

While gorillas and other Old World primates (e.g. baboons) exhibit relationships between males and infants that have fitness consequences for one or both participants, such relationships occur in their most extreme and elaborate form in humans. In humans, male care is facultative rather than obligatory^13^, but some level of male investment in offspring is a cultural universal^49,50^. Despite this, morphological characteristics and behavior among extant humans, as well as fossil hominin remains, suggest that monogamy was not the predominant state for much of our evolutionary history^51^. The fact that male care occurs (if to varying degrees) in several non-monogamous Old World primate species, including humans, suggests that male care can evolve via some alternative selection route. The data presented here provide empirical evidence for a scenario in which males’ interactions with infants, and their mating effort, could complement one another instead of working at cross purposes. This pattern could initially catalyze social bond formation between males and young, despite the assumed evolutionary fitness costs these relationships would have imposed.

## Methods

### Ethics statement

This research was strictly observational. Behavior data and fecal sample collection protocols followed the relevant guidelines put in place by the Rwanda Development Board, the Dian Fossey Gorilla Fund, and the Max Planck Institute for Evolutionary Anthropology.

### Subjects and social groups

All data were collected on the mountain gorillas monitored by the Dian Fossey Gorilla Fund’s Karisoke Research Center in Volcanoes National Park, Rwanda. During behavioral data collection in 2003 and 2004, the gorillas lived in three multi-male/multi-female groups containing 24–58 individuals, each of which contained between 7 and 9 adult males. During focal animal follows, we recorded the occurrence and duration of all grooming and resting in contact events between males and infants.

The dataset contains 23 adult males who were at least 9 years old at the midpoint of behavioral data collection. While they do not reach full size until ~15 years of age^35^, males as young as 8 years have sired infants in this population^26^. To be included in our analyses, males needed to have at least 10 hours of focal follow data available, though most had more (mean observation hours/animal = 20.92, SD = 8.65, range = 10.42–38.38). The data hours were amassed over a minimum of 12 50-minute focal follows, across the course of at least 12 of the 18 months during which data were collected. More than 12 individual follows were usually necessary to account for out-of-view time, and most subjects were followed at least once in all 18 of the study months. We removed two young males (8 and 9 years old respectively at the behavioral data midpoint) from the analyses because of insufficient data. As of 2014, the last year for which paternity data were available, neither of these males was known to have sired any infants. We also removed two other males with insufficient behavioral data, who dispersed from our study population during behavioral data collection at the ages of 15 and 16. One of these males was known to have sired 2 infants as of 2014, both with females who did not live in his group in 2003–04. The other has no known infants.

As of the time of writing, 16 of the males (70%) were confirmed dead. Four (17%) dispersed from their natal groups before 2010 and are believed to be either dead or solitary silverbacks. One of these disappeared in the midst of clear health problems. The other three were occasionally encountered as lone silverbacks in the years immediately preceding their dispersals, but have not been observed for at least five years, and were not detected during a 2015 genetic census of the Virunga National Park mountain gorilla population (pers. comm., A–C Granjon; Karisoke long-term records). Two other animals, 24 and 26 years old, have been regularly observed living as solitary silverbacks since their natal dispersals. Genetic paternity data have confirmed extremely low rates of extra-group paternity in this gorilla population (Karisoke long-term records^26^), so it is unlikely the males who dispersed and became solitary silverbacks sired large numbers of infants that are not included in our paternity data. The one remaining male, age 22, is the dominant male in his social group as of the time of writing in 2018. He was a subordinate male during behavioral data collection in 2003–04.

### Paternity determination

Our outcome variable of interest was the total number of known infants that a male sired by 2014, the latest year for which paternity data were available. Paternity was assigned using non-invasive methods (described in^26,52^). We collected a minimum of three fecal samples from each infant, mother, and potential father (assumed to be any male in the social group age 7+^25^). Samples were collected by Karisoke employees and researchers who were tested on their ability to identify each animal before beginning data collection. We extracted DNA and genotyped each sample at either 16 or 19 autosomal microsatellite loci, including replication to prevent errors like allelic dropout. We confirmed sample identification by comparing either known mother/infant pair genotypes, or at least two samples purported to be from the same gorilla. We also used a PCR-based sexing assay to confirm reported sex^53^. To assign paternity, we required a 95% confidence level using CERVUS 3.0.3^54^.

Males sired a mean of 4.74 infants each (SD = 6.94). The dataset analyzed here includes 109 infants born between 1985 and 2014, which is 84% of the infants of known paternity (n = 130) born in this period. The rest were excluded because we did not have behavioral data from their fathers due to death or dispersal. We cannot conclusively determine the exact number of infants each male sired because some infants died before samples could be collected. There is also a small possibility that a male sired infant(s) with female(s) outside the study groups. However, undetermined paternities should be distributed randomly, thereby adding noise but not bias to our data.

### Behavioral data

For each adult male focal subject, we calculated the total percentage of focal follow time he was engaged in resting in physical contact with infants (individuals <3.5 years old^55^) and grooming with infants. Measures were direction-agnostic. In general, infants are more likely to initiate interactions with males than males are with infants^3^, but both of these types of affiliation require physical touch, a high degree of tolerance, and may potentially interfere with other activities for both of the involved animals. Though such behavior should not be considered investment until and unless any costs are empirically established^3,24^, it would nonetheless be evolutionarily relevant behavior if it is related to reproductive outcomes.

Grooming and resting in contact rates are non-independent and correlated for this demographic^3^. We therefore used a composite measure of the two to reduce the risk of committing a Type I error^56^. Following a widely-used method (described in^57^), for each of the two behaviors, we divided the value for an individual male by the mean value of all the males. Next, we summed the numbers generated for each of the two components for each male, and divided by 2, to obtain a composite affiliation value. For these composite values, higher numbers mean that the male in question spent more time grooming and resting in contact with infants than the average male did, and lower numbers mean that the male spent less time grooming and resting in contact with infants than the average male did. The composite number for each male was the affiliation predictor used in the statistical models.

Anecdotally, males’ behavior towards infants is quite stable over time (pers. obs., Karisoke long-term records). While many studies rely on an underlying assumption that intra-individual behavior is relatively consistent across time, to confirm that our data were representative of males’ behavior over longer periods, we tallied the same composite measure for seven of the males in the study for whom we had identical behavioral data collected in 2011 and 2012. For these males, the correlation between the 2003–04 composite scores and the 2011–12 composite scores was 0.325. We interpret this as a high degree of behavioral stability in light of the considerable rank and group composition changes that all males experienced in the intervening seven years^21^.

In addition to determining how much time males spent grooming and resting in contact with infants, we also calculated the proportion of time each male had at least one infant <2 m away, a commonly used distance of close proximity in this species^58–60^. If females are attracted to reproductively successful males, this could lead to correlations with infant affiliation simply because infants tend to stay close to their mothers. To assess this possibility, we compared the results of a model that included the proportion of time that males had an infant within 2 m (proximity) to the identically structured model that contained the affiliation (grooming and resting in contact) predictor. If the model results are similar for both predictors (proximity and affiliation), this suggests any relationship between affiliation and reproductive success may be driven by males’ proximity to mothers and infants. If the results are different, then proximity is less likely to explain the relationship between affiliation and reproductive success.

### Control variables

Because older males necessarily have had more time to sire infants than younger ones, we controlled for males’ ages, calculated as of the time they exited our dataset (i.e., at the point where there were no longer paternity data available for them). We considered males to have exited if they either died, or dispersed and were no longer monitored by Karisoke. The exit age for males who remained in the study population throughout was their age as of December 31, 2014, the last year for which paternity data were available. Across all males, exit age range = 14–38 years, mean = 23.22, SD = 6.62. This variable also functionally serves as a control for differences in male longevity. Second, infants are known to affiliate more with older males (who are typically, but not always, more dominant) than younger ones^3,4,24^. To control for this, we included the age of the male at the midpoint of behavioral data collection (9–30 years; mean = 15.83, SD = 6.42).

We also included two rank-related controls. Dominance rank is strongly correlated with reproductive success for males in this population^26^, though with considerable temporal variation in the degree of reproductive skew^24^. Infants are also known to interact more with males of higher rank^3,24^. To account for both of these factors, we included the males’ ranks at the time behavioral data were collected, and the highest dominance rank the male achieved before he exited the dataset. Ranks were determined using displacement data (where one animal moves out of another’s way as it approaches), a well-established method used for gorillas and many other primates^23^. Males who held a rank lower than gamma were all classified as one category, subordinate, since displacement data among lower-ranking males is usually insufficient to conclusively determine their place in the dominance hierarchy^23^. Rank was coded as a categorical variable where 0 = alpha rank, 1 = beta, 2 = gamma, and 3 = subordinate.

In addition to the age and rank controls, we included the total potential infants a male could have sired as an exposure variable, to account for differences in reproductive opportunity associated with different group sizes and availability of females during fertile windows. We operationalized a male’s siring potential as the total number of infants born in his social group(s), from the time he was 9 years old until 9 months after he exited the data set (min = 8, max = 104, mean = 34.65 possible infants sired). This calculation accounts for gestation time, and assumes a male could have started siring infants when he was as young as 8 years old^24,26^ and continued siring infants up until exiting the dataset.

Another potentially relevant control was the number of offspring a male had physically present during behavioral data collection. It is possible that males who had more offspring in total had more offspring present during behavioral data collection, in which case one reasonable inference is that the males who had the most offspring also interacted the most with infants (i.e., males who had the most offspring available simply did more ‘parenting’). In our exploratory analyses, we found that this variable was not a statistically significant predictor of reproductive success, nor did it change the relationship between the outcome variable and the predictor of interest (affiliation), so we excluded it from the models reported here to avoid overfitting.

### Data analysis

Visual inspection and summary statistics confirmed significant overdispersion of our outcome variable (mean = 4.739, variance = 48.11), so we used negative binomial regression models with robust standard errors to correct for minor violations of underlying assumptions^61^. Due to the known strong relationship between dominance and reproductive success, as well as infants’ preferences for associating with high-ranking males^24^, we ran the model twice. The first contained the full complement of males (n = 23). The second contained only the males who held beta or lower dominance ranks during behavioral data collection (n = 20). Since males of different ranks may employ different types of reproductive strategies^62^, the reduced model was used to help us determine whether the dominant males might either drive, or obscure, a relationship between male-infant affiliation and male reproductive success. With the exception of the removal of the dominant males (n = 3), the two models were structured identically. All analyses were run using Stata 13 (StataCorp, College Station TX).

## Data Availability

Data used in this manuscript are the property of the Dian Fossey Gorilla Fund. All data and Stata code used in these analyses are available upon reasonable written request directed to the corresponding author.
